# Artificial Intelligence in Mental Health Services Under Illinois Public Act 104‐0054: Legal Boundaries and a Framework for Establishing Safe, Effective AI Tools

**DOI:** 10.2196/84854

**Published:** 2025-12-04

**Authors:** Daniel Szoke, Sarah Pridgen, Philip Held

**Affiliations:** 1 Department of Psychiatry and Behavioral Sciences, Rush University Medical Center, 1645 W Jackson Blvd, Suite 602, Chicago, IL, 60612, United States

**Keywords:** artificial intelligence, mental health, psychotherapy, legislation as a topic, evidence-based practice, health policy

## Abstract

Artificial intelligence (AI) applications in mental health have expanded rapidly, and consumers are already using freely available generative AI models for self-guided mental health support despite limited clinical validation. In August 2025, Illinois enacted Public Act 104‐0054, the first state statute in the United States to explicitly define and regulate the use of AI in psychotherapy services, establishing boundaries around administrative support, supplementary support, and therapeutic communication. While the Act clarifies several aspects of AI use in therapy, it also leaves important gray areas, such as whether AI-generated session summaries, psychoeducation, or risk-flagging functions should be considered therapeutic communication. Drawing on the history of empirically supported treatments in psychology, we argue that a framework of evidence, safety, fidelity, and legal compliance could help determine when AI tools should be integrated into clinical care. This approach provides a concrete pathway for balancing patient protection with responsible innovation in the rapidly evolving field of mental health AI tools.

## Introduction

Artificial intelligence (AI) applications in mental health have expanded rapidly in recent years [1,2]. Some early-stage generative AI-based systems have been shown to be capable of detecting symptoms of mental illness, creating evidence supported treatment plans, and even engaging in therapeutic conversations [2,3]. However, evidence concerning “off-the-shelf” models (eg, OpenAI’s ChatGPT or Anthropic’s Claude) that were given simple prompts without examples or additional training, known as zero-shot approaches, have been found to be limited in their diagnostic accuracy and engagement with users on cultural and emotional topics [4,5]. Meanwhile, consumers are already using these freely available models for self-guided “therapy” despite limited clinical validation, with a plurality of the public believing that AI should play a role in providing mental health support [6,7]. Despite stated benefits for some, the use of generative AI tools in mental health contexts has also produced negative, unintended outcomes [4,8] and led to calls for further regulation from bodies including the American Psychological Association (APA) [9]. While increasing access to mental health services is essential given existing shortages, and the development of generative AI tools may be one part of the solution, the implementation of these tools in clinical contexts must be guided by rigorous evidence and ethical safeguards.

Policymakers have begun to respond to these calls for action. In August 2025, Illinois enacted Public Act 104‐0054, the first state statute in the United States to explicitly define and regulate the use of AI in psychotherapy services [10]. The act introduces specific definitions, delineates prohibited uses, and requires written, specific, and revocable consent when AI processes session data. In this article, we aimed to first clarify what the law allows and prohibits, and second, identify areas of ambiguity that may benefit from future clarification. One area in need of further exploration is a set of empirical standards that current and future AI tools should meet before being used in mental health treatment. Our final aim is to suggest a potential standard based on previous work in the field of psychology.

## Public Act 104-0054 at a Glance

### Overview

The Act [10] applies to psychotherapy services delivered within Illinois and establishes a statutory framework for the use of AI in these contexts [10] (see the study by Shumate et al [11] for a recent review of AI laws in all 50 states). The statute regulates three types of AI use in psychotherapy including *administrative support*, *supplementary support*, and *therapeutic communication*. Below, we will provide a summary of the Act, but we invite readers to review the Act themselves, especially those practicing under its jurisdiction.

### Administrative Support

Administrative support includes scheduling and/or reminders of appointments, processing billing and insurance claims, and drafting general communications related to therapy that specifically do not include “therapeutic advice.” Potential examples could include using AI to write a standard welcome letter for patients who are starting therapy with a new provider or using AI to review patients’ health insurance benefits and provide plain language summaries (eg, “Based on their current coverage, Patient X will owe a US $20 dollar co-pay for a standard, 50-min psychotherapy session”). The provider still accepts full responsibility for the output, and any data shared with AI.

### Supplementary Support

Supplementary support includes maintaining patient records, including preparing therapy notes, and analyzing data to track patient progress (if data are anonymized and reviewed by a licensed professional). Importantly, supplemental support tasks that use recordings or transcripts of therapy sessions are subject to explicit patient informed consent. If a patient consents, potential examples of supplementary support could include using AI to draft session notes based on session transcripts, subject to review by the therapist. However, consent is not needed to conduct a supplementary task such as generating graphs of patient progress in treatment based on anonymized data from self-report measures (eg, Patient Health Questionnaire-9 scores).

### Therapeutic Communication

Therapeutic communication is defined as “any verbal, non-verbal, or written interaction conducted in a clinical or professional setting that is intended to diagnose, treat, or address an individual’s mental, emotional, or behavioral health concerns” (Public Act 104‐0054, 2025 § 10) [10]. The act gives several examples; however, it notes that the definition is not limited to the examples provided. Examples of therapeutic communication include directly interacting with patients to understand or reflect their thoughts, emotions, or experiences; providing guidance or therapeutic strategies; offering emotional support or empathy; developing goals or treatment plans; and offering feedback about the patient’s behaviors to promote psychological growth.

### Enforcement

Public Act 104‐0054 is notable for its definitions and enforceable provisions, which distinguish it from more general or nonbinding AI guidelines provided by professional organizations like the APA [12]. Violations, enforced by the Illinois Department of Financial and Professional Regulation, of the statute may result in civil penalties, with fines of up to US $10,000 per violation. However, the law does not fully clarify how certain AI functions, such as generating psychoeducational materials or asynchronous mood check-ins, should be classified. This leaves a set of “gray area” applications where legal compliance may be uncertain and professional judgment becomes essential.

### Clearly Permitted, Clearly Prohibited, and Gray Areas

Public Act 104‐0054 distinguishes between AI functions that are clearly permissible or clearly prohibited, but similar to other laws that attempt to regulate cutting edge technologies, the Act does not address many potential applications that fall into areas of legal ambiguity (Table 1). For example, it may be permissible for a clinician to provide session transcripts to an AI tool that was purpose-built (ie, specifically trained and tested) to write a session summary for the patient, as long as the patient has provided informed consent. Research has established that existing off-the-shelf AI models like Google’s Gemini Pro can assist with basic session documentation [13], so although not yet tested, a patient summary feature is likely not far. Furthermore, off-the-shelf models are already performing decently well, but we assume that purpose-built mental health AI tools will perform even better. These high-performing tools could generate summaries that include a description of potential homework assignments such as, “Complete one thought record each day on a difficult thought that you encountered that day.” This use of AI may raise several questions: Is the description of the homework assignment a therapeutic communication? What if it is a direct quote from the therapist, repeated in text generated by AI? Taking the example further, it has been shown that querying patients via text messages about therapy homework completion is linked to greater compliance, but only when the patient receives praise in response to their completion [14]. If a clinician leverages an evidence-based AI tool to query patients and provide praise, has the AI engaged in a therapeutic communication? Until these questions are addressed, providers do not have a clear path for the implementation of seemingly basic, yet possibly beneficial, uses of AI in mental health services.

**Table 1.** Artificial intelligence (AI) use in therapy under Illinois Public Act 104‐0054.

Category	Examples	Notes
Allowed	All administrative support including appointment scheduling; reminders; billing and insurance processing; maintaining patient records; analyzing anonymized data for trends; and identifying and organizing external resources or referrals	These functions must remain administrative or supplementary. A licensed clinician retains the full responsibility for care. Written consent is required if session recordings or transcripts are used for AI processing.
Prohibited	Making independent therapeutic decisions; directly engaging in therapeutic communication with patients; and detecting emotions or mental states	These actions are explicitly banned regardless of patient consent.
Gray area	AI-generated psychoeducation or session summaries that could be interpreted as therapeutic advice; AI safety tools that alert a therapist to patient phrases in session that may be related to risk of self-harm; “check-in” AIs that query mood or well-being; and AI-aided intake tools that straddle administrative and diagnostic functions	These functions may be permissible if they are clearly supplementary and reviewed by a clinician. The law does not fully delineate their boundaries, creating potential compliance uncertainty.

While the previous example focused on homework completion, higher stakes mental health tasks such as suicide risk detection and management should also be considered. Research groups have suggested the potential benefits of AI in the detection of suicidal risk based on features like patients vocal tone or language used in therapy sessions [15,16]. If, following patient consent, a purpose-built AI tool was able to listen to live or recorded therapy sessions and flag the clinician to conduct additional risk assessment based on the patient’s voice or word choice, does that mean the AI tool detected an emotional state? Is labeling a patient as “potentially in need of additional risk assessment” the same as “making an independent therapeutic decision”? Again, without further clarification, providers will struggle to implement such tools, even though such tools could objectively increase the safety of mental health consumers.

As written, the Act may also have adverse effects on clinical research. For example, the current law does not provide an explicit exemption for research on applications of AI in mental health. Thus, depending on the interpretation of the Act, the absence of a formal exemption may prevent researchers from exploring the potential effectiveness and safety of these tools.

We pose these hypothetical examples to illustrate that while definitions provided thus far are helpful in establishing initial guidelines, there are significant gaps that remain for future adjudication. As a final area to consider, we question whether strict prohibitions on therapeutic communication should apply to purpose-built AI tools that have established a strong evidence base for the safety and improvement of a patient’s mental health. While clearly prohibited under the current guidelines, we propose that a future AI tool that manages to establish strong empirical support should be allowed for use by consenting patients. With the knowledge that patients are currently engaging with non-evidence-based “off-the-shelf” models for psychological guidance [6,7] and the harms this may cause [9], the field should take seriously the need for standards of evidence to judge future mental health AI tools.

## Toward Empirically Supported Use of AI in Mental Health

What is missing from Public Act 104‐0054 is a mechanism for determining whether a given tool is safe and effective. The absence of such a pathway may hinder advancement in the area of mental health AI tools that could meet both regulatory and professional standards. This may also create uncertainty for clinicians seeking to integrate AI into practice in the future.

When we examine the history of clinical psychology, we can identify developments related to the safety and effectiveness of empirically supported practices. Similar frameworks could function as examples for establishing mental health AI tools as safe and effective. In the 1990s, in response to the growing number of potentially effective psychotherapies, a question arose concerning which techniques should be taught in training programs and used in practice. Through that decade, APA’s Division 12 (Society of Clinical Psychology) appointed a taskforce aimed at establishing a standard of evidence for psychological treatments, to determine which practices had established adequate evidence of safety and efficacy [17]. This led to the establishment of Chambless and Hollon’s [18] definition for empirically supported treatments (ESTs). In brief, they define “well-established” treatments as those with two or more independent randomized controlled trials (RCTs) or strong single-case designs that show the manualized treatment was superior or equivalent to an already established treatment. They define a treatment as “possibly efficacious” if only one RCT has been conducted, or if multiple non-independent (ie, conducted by the same team of researchers) studies have been conducted. These standards, while not without their criticisms [17], led to several positive changes. First, because researchers in the field had a definition to go by, the establishment of standards coincided with a proliferation of RCTs that aimed to meet the Chambless and Hollon definition of evidence [19,20]. Second, training and practice guidelines set by the APA have directed training programs and providers to practice treatments that have met the standards of evidence, meaning clinicians could base both treatment and training decisions on a standard of evidence rather than a historical preference or orientation of a specific training program [21]. Furthermore, these programs can teach students the standards for empirically supported treatments, meaning students will be prepared to make independent decisions about which practices to employ for the rest of their careers. Perhaps most importantly, as noted by Tolin and colleagues [20], the standards have provided protection to consumers of therapy services, who can now be educated on which treatments have an evidence base, and seek out specific treatments based on accumulated evidence.

As the use of AI in mental health continues to evolve (as shown by Guo et al [22] in a recent systematic review), we see similar gaps that may benefit from a similar set of standards. Systematic empirical research on the safety and efficacy of AI tools for mental health will be essential for determining whether these tools are effective, for whom they are most helpful, and under what conditions they should be used. This work will also clarify the contexts in which AI tools introduce meaningful risks, enabling those risks to be more accurately characterized and proactively addressed. Currently, researchers do not have a standard to aim for when conducting research on new AI applications in mental health. Programs currently training the next generation of clinicians, who will be the first to practice their entire careers in a post-AI world, have no standards to follow when selecting which AI tools students should learn to implement in their practice. Finally, consumers are left unprotected, having no way to discern which AI tools have established a base of evidence verses those that have not. This leads to the necessity for laws like Public Act 104‐0054, a blanket ban on certain uses of AI, because there is no established standard of evidence. This is also an opportune time to develop a standard of evidence, as mental health AI tools are just now being developed. While therapy techniques had been established and practiced for decades by the time of Chambless and Hollon’s definition in 1998, AI developers can plan and design tests of their tools prospectively with a set of criteria in mind.

We propose a set of guidelines below, modeled largely after Chambless and Hollon’s [23] original definition of evidence supported treatments. Before presenting these criteria, it is important to acknowledge that a newer set of Division 12 standards emerged in 2015 [20]. The standards proposed by Tolin et al [20] acknowledged the extensive proliferation of RCTs (a number that is still increasing [19]), and notable concerns that the quality can vary from one RCT to the next (a trend that has also continued [24]). These newer standards moved beyond Chambless and Hollon’s criteria of two RCTs and/or single case designs and instead asked those reviewing treatments for potential status as “empirically supported” to review all available evidence, the quality of the evidence (including risk for bias), and grant designations of “very strong,” “strong,” or “weak” support. The added nuance and emphasis on stronger evidence, such as meta-analysis, are an excellent future goal for the criteria of AI in mental health. However, we argue that as tests of AI in mental health are still in their infancy, we should model initial criteria off of those established in Chambless and Hollon’s work [18], with the eventual goal of advancing the standards to match Tolin and colleagues’ definitions [20] as evidence amasses.

First, AI tools should demonstrate their efficacy and safety, similar to Chambless and Hollon’s standard of efficacy [18]. Two or more RCTs or strong single-case designs that demonstrate superiority or equivalence to an established treatment should be required. An additional specification should be that these studies demonstrate an absence of adverse or harmful effects. A hypothetical RCT could compare cognitive processing therapy for post-traumatic stress disorder (PTSD) to cognitive processing therapy for PTSD with an adjunctive AI tool used to help with homework assignments.

Second, Chambless and Hollon [18] described a need for the manualization of treatment, in part to establish internal validity. In the case of AI tools for mental health, this can be explored as fidelity to the intended function. In treatment outcome literature, while not without its criticisms, a rule of thumb of 80%‐100% fidelity to the intended intervention is considered “high integrity” [25]. The same should be applied to test for the fidelity of an AI tool. For example, if an AI tool is used to generate a mood check-in for patients and respond with an empathic statement, several hundred sample conversations could be generated, and then randomly screened for fidelity by expert clinicians. If 80%‐100% of the time, the AI tool provided an appropriate mood check-in and empathic response, it would be considered meeting the fidelity benchmark.

Third, Chambless and Hollon [18] emphasized the importance of identifying in which populations and settings the treatment was tested. The same can be applied for AI tool implementations in mental health. For example, an AI tool designed to assist with exposure focused homework might function well with adult patients in outpatient specialty clinics but perform poorly in primary care, inpatient settings, or with adolescents. Likewise, the clinical presentation of the population matters. An AI tool that reliably supports patients with depression may not be generalizeable to individuals with psychosis, PTSD, or complex comorbidities. Special attention should be paid to language, cultural factors, and digital literacy, as these elements can affect both engagement with the tool and the accuracy of the AI tool’s outputs.

Finally, we add that any use of AI should provide proof of its adherence to laws, such as Public Act 104‐0054 and the Health Insurance Portability and Accountability Act (HIPAA). For instance, an AI note-drafting tool that ingests session transcripts would need to show that transcripts are stored securely, are only accessible to authorized clinicians, and are deleted in accordance with retention policies. Equally important, the system must include a clear and understandable consent process for patients, detailing what data will be processed, how it will be used, and under what circumstances it might be shared. Compliance should also include mechanisms for auditing and updating AI systems as laws evolve, given that regulatory standards in this area are likely to change rapidly. Just as manualized treatments must be updated when diagnostic criteria or clinical standards shift, AI developers and clinicians should treat legal and regulatory adherence as a dynamic, ongoing requirement rather than a one-time hurdle (Table 2).

**Table 2.** Chambless and Hollon (1998) EST^a^ criteria for therapy versus modified criteria for artificial intelligence (AI) tools.

Criteria	Original Chambless and Hollon (1998) EST criteria	Modified criteria for AI tools under Public Act 104‐0054
Efficacy, effectiveness, and safety	≥2 independent randomized controlled trials (RCTs) or strong single-case designs demonstrating superiority or equivalence to an established treatment	Same standard of evidence, plus empirical demonstration of no harm
Manualization and fidelity of intervention	A treatment manual specifying content and delivery to ensure fidelity	Demonstration that the AI consistently adheres to intended functions and prompts, with fidelity.
Defined population/setting	Clear description of the populations and settings in which the treatment was tested	Clear description of the patient groups and contexts in which the AI tool has been tested
Legal and privacy compliance	—^b^	Meets HIPAA and legal statute requirements, including consent requirements and secure handling of protected health information

a EST: empirically supported treatment.

b not applicable.

A challenge here is that underlying systems may change frequently, and it may be difficult to determine when new functionality, safety, and security checks are needed. For example, if a purposefully developed AI tool is built on top of Meta AI’s LLaMA 3, it may be necessary to retest the tool when updating to LLaMA 4. However, what about a smaller change, such as when the company providing the AI system makes an update that leads to longer response times from the tool or adjusts the word choices in the responses? These kinds of behind-the-scenes updates, often called “patches to the application programming interface,” may subtly affect the users experience of the tool. Even if the change seems minor, it could alter whether the tool performs the intended function, raising the question of whether a new round of fidelity testing is needed. As a parallel, empirically supported treatments such as cognitive processing therapy for PTSD often release updated manuals with slightly altered guidance and/or materials for the therapeutic techniques [26,27]. While the entire manual is not retested for efficacy, the changes are assumed to be based in new evidence gathered and expert opinion. Perhaps similar standards could be established in the future to address the frequently changing nature of AI tools.

## Conclusions

The rapid expansion of AI in mental health care creates both opportunities and regulatory challenges. Illinois Public Act 104‐0054 is among the first state laws to set explicit legal boundaries for AI use in therapy, offering detailed definitions and enforceable prohibitions alongside consent requirements [10]. While these provisions clarify certain aspects of practice, they leave significant uncertainty in areas where AI functions may be interpreted as either supplementary or therapeutic.

Here we attempted to address a set of guidelines for the use of AI with consenting adults; further work should establish specific guidelines for children who may be particularly at risk of harm when using AI unsupervised [28]. Additionally, questions remain about the level of change to an AI tool that would require the re-establishment of empirical support; however, we note that the field does not have an established standard of re-evaluating psychotherapy techniques after updates are made either.

Although these areas of uncertainty create practical challenges, we recognize that some degree of ambiguity is often intentional and even necessary in early technology legislation. Broad statutory language may be used here so that the law can remain relevant as tools evolve, and to allow regulatory agencies to work out technical details. This flexibility gives the Illinois Department of Financial and Professional Regulation and future case law room to refine how the statute should operate in real-world settings, but it also underscores the need for structured guidance to help clinicians and developers navigate these gray areas responsibly. Our proposed adaptation of the EST framework offers one concrete pathway for developing such guidance by articulating standards of effectiveness, fidelity, and legal compliance that could inform future administrative rules and professional guidelines. States considering similar legislation could adopt both the regulatory structure of Public Act 104‐0054 and a definition of empirically supported AI tools, thereby pairing statutory boundaries with operational criteria. In parallel, future research should take a socio-legal approach and examine how AI tools are actually being implemented in Illinois once the law has been in effect long enough to generate meaningful patterns of use, as such data could further reveal areas in need of clarification. Collaboration among policymakers, professional associations, and researchers will be essential to refine these evidence-based standards and ensure that patient protection and technological innovation advance together.

## Abbreviations:

AI: artificial intelligence
APA: American Psychological Association
EST: empirically supported treatment
HIPAA: Health Insurance Portability and Accountability Act
PTSD: post-traumatic stress disorder
RCT: randomized controlled trial
